# *Talaromyces marneffei* Genomic, Transcriptomic, Proteomic and Metabolomic Studies Reveal Mechanisms for Environmental Adaptations and Virulence

**DOI:** 10.3390/toxins9060192

**Published:** 2017-06-13

**Authors:** Susanna K. P. Lau, Chi-Ching Tsang, Patrick C. Y. Woo

**Affiliations:** 1 Department of Microbiology, Li Ka Shing Faculty of Medicine, The University of Hong Kong, Pokfulam, Hong Kong; microbioct@connect.hku.hk; 2 State Key Laboratory of Emerging Infectious Diseases, The University of Hong Kong, Pokfulam, Hong Kong; 3 Research Centre of Infection and Immunology, The University of Hong Kong, Pokfulam, Hong Kong; 4 Carol Yu Centre for Infection, The University of Hong Kong, Pokfulam, Hong Kong; 5 Collaborative Innovation Centre for Diagnosis and Treatment of Infectious Diseases, The University of Hong Kong, Pokfulam, Hong Kong

**Keywords:** *Talaromyces marneffei*, genomics, transcriptomics, proteomics, metabolomics

## Abstract

*Talaromyces* *marneffei* is a thermally dimorphic fungus causing systemic infections in patients positive for HIV or other immunocompromised statuses. Analysis of its ~28.9 Mb draft genome and additional transcriptomic, proteomic and metabolomic studies revealed mechanisms for environmental adaptations and virulence. Meiotic genes and genes for pheromone receptors, enzymes which process pheromones, and proteins involved in pheromone response pathway are present, indicating its possibility as a heterothallic fungus. Among the 14 Mp1p homologs, only Mp1p is a virulence factor binding a variety of host proteins, fatty acids and lipids. There are 23 polyketide synthase genes, one for melanin and two for mitorubrinic acid/mitorubrinol biosynthesis, which are virulence factors. Another polyketide synthase is for biogenesis of the diffusible red pigment, which consists of amino acid conjugates of monascorubin and rubropunctatin. Novel microRNA-like RNAs (milRNAs) and processing proteins are present. The dicer protein, dcl-2, is required for biogenesis of two milRNAs, *PM-milR-M1* and *PM-milR-M2*, which are more highly expressed in hyphal cells. Comparative transcriptomics showed that tandem repeat-containing genes were overexpressed in yeast phase, generating protein polymorphism among cells, evading host’s immunity. Comparative proteomics between yeast and hyphal cells revealed that glyceraldehyde-3-phosphate dehydrogenase, up-regulated in hyphal cells, is an adhesion factor for conidial attachment.

## 1. Introduction

**Figure 1:**
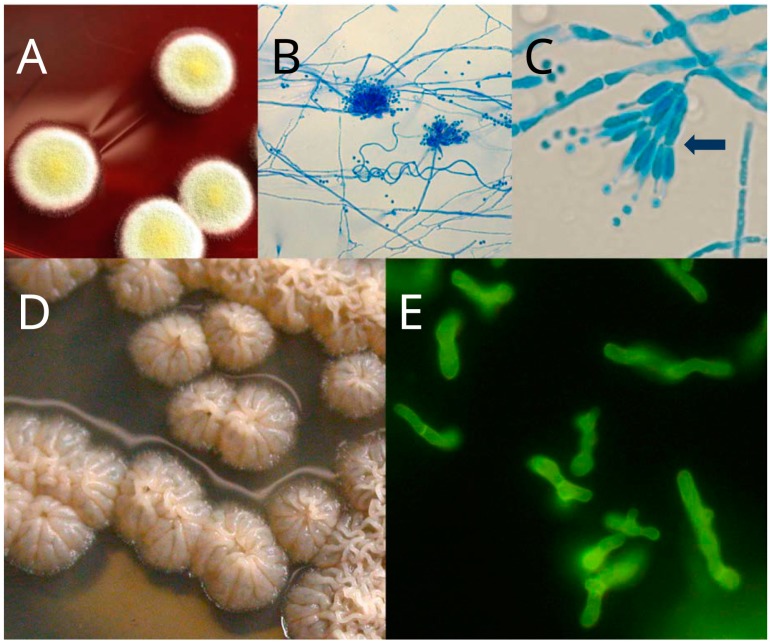
Thermal dimorphism of *Talaromyces marneffei*: (**A**) at 25 °C, *T. marneffei* grows as a mold that produces greenish-yellow to yellow conidia and secretes a characteristic diffusible red pigment; (**B**) microscopically, hyphae are twisted; (**C**) conidiophores are mostly biverticillate (arrow) and resemble those of *Penicillium* species, while conidia are globose to sub-globose and are generated from phialides; (**D**) at 37 °C, *T. marenffei* grows as a yeast with a cerebriform colony appearance and no diffusible red pigment is produced; and (**E**) yeast cells are divided by budding. Microscopic slides were prepared using the stains (**B**,**C**) lactophenol cotton blue or (**E**) calcofluor white with 5% potassium hydroxide and observed using the Eclipse Ni-U upright microscope system (Nikon, Tokyo, Japan).

*Talaromyces marneffei* (Segretain et al.) Samson et al. is a thermally dimorphic fungus. This fungus exhibits yeast-like morphology at 37 °C, while, at 25 °C, it grows as a mold that produces a characteristic diffusible red pigment (Figure 1). *T. marneffei* was for the first time isolated from *Rhizomys sinensis* (Chinese bamboo rats) in 1955 [1], initially as *Penicillium marneffei*. Subsequently, this fungus was also recovered from other bamboo rat species belonging to the *Rhizomyinae* Subfamily, such as *Rhizomys pruinosus* (hoary bamboo rats), *Rhizomys sumatrensis* (large bamboo rats), and *Cannomys badius* (lesser bay bamboo rats) [2,3,4]. In 2011, based on molecular data, which included the RNA polymerase II largest subunit gene (*rpb1*) sequence and internal transcribed spacer (ITS) region sequence, the fungus was reclassified under the genus *Talaromyces* [5]. 

*T. marneffei* is endemic in Southeast Asia, and most patients with *T. marneffei* infections either are residing in or have travel histories to Southeast Asia [6]. The fungus is believed to be taken up through inhalation of fungal spores [7]. It infects mainly cells of the reticuloendothelial system [8]. Patients with *T. marneffei* infections are mostly immunocompromised. Traditionally, it mainly infects HIV-positive patients and it is an important AIDS-defining condition for patients in Southeast Asia [6]. Recently, due to advancement in both technologies in diagnosing various primary immunodeficiency states and medications which result in generation of more secondary immunodeficiency conditions, *T. marneffei* is found to be causing infections in other groups of patients, such as those with antibodies against interferon gamma and patients receiving monoclonal anti-CD20 antibodies or inhibitors to kinases [9]. Occasionally, *T. marneffei* infection is also observed in patients with other immunocompromised conditions, such as systemic lupus erythematosus, bone marrow and solid organ transplant recipients, various malignancies, T-lymphocyte-depleting immunsuppressive drugs recipients, etc. [10]. 

In 2011, we generated the draft genome of an isolate of *T. marneffei* (PM1) recovered from a patient in Hong Kong [11]. In the past six years, using the draft genome sequence as well as performing aedditional transcriptomic, proteomic and metabolomic studies, we analyzed the various characteristics of this fungus. In this article, we review the potential mechanisms for environmental adaptations and virulence based on these studies.

## 2. Mitochondrial Genome and Phylogeny

**Figure 2:**
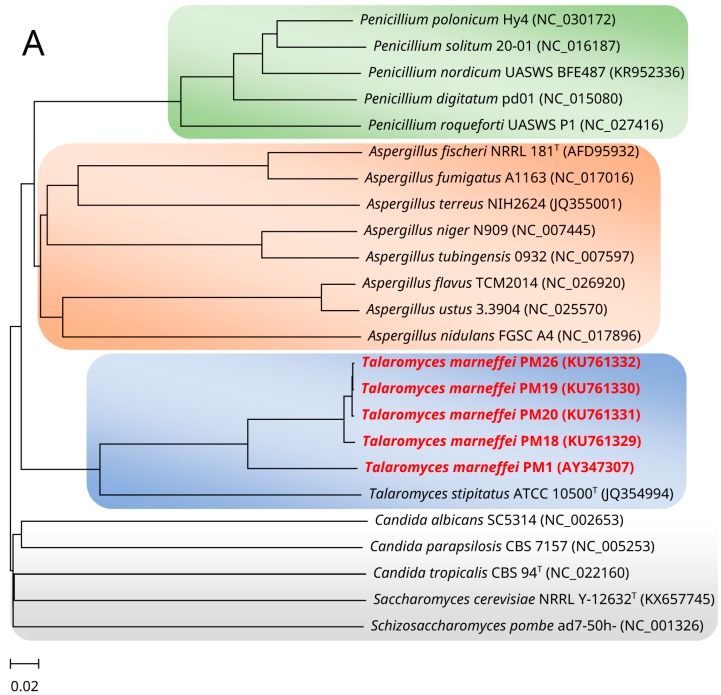

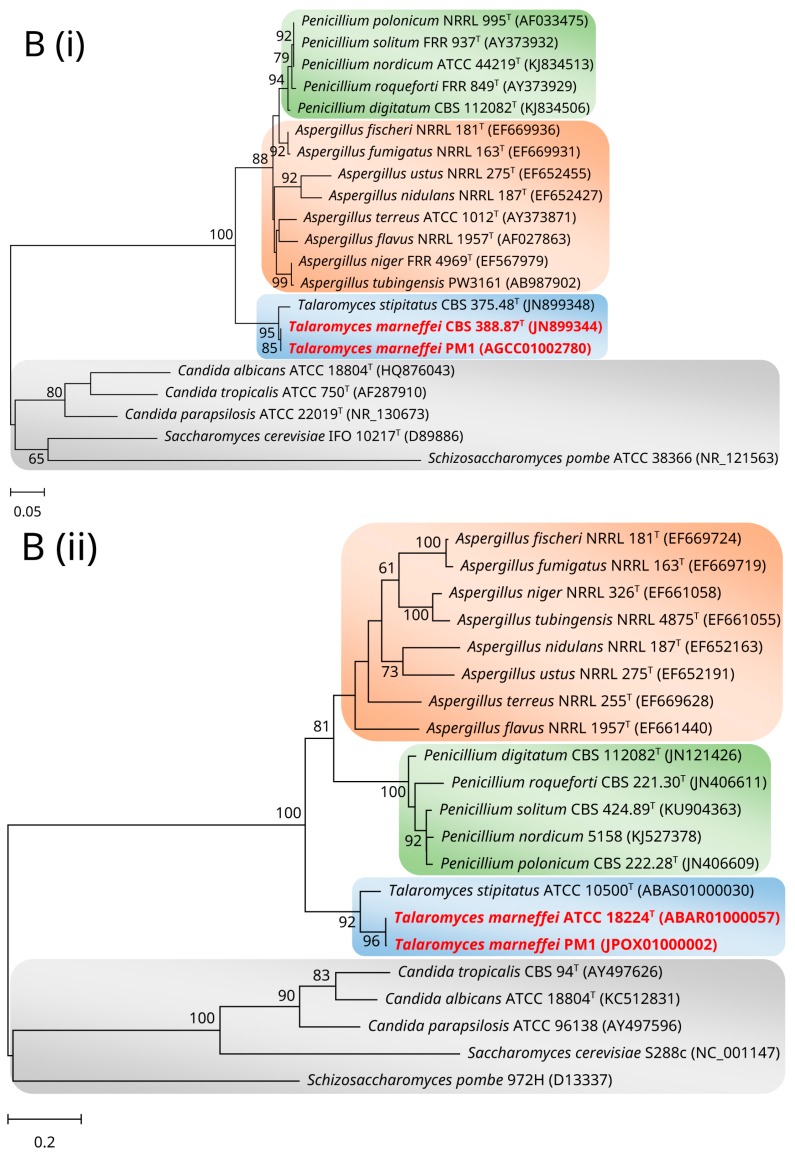
Phylogeny of *Talaromyces marneffei*. (**A**) Phylogenetic tree showing the relationship of *Talaromyces marneffei* with other molds and yeasts, inferred from all the coding sequences within the complete mitogenomes of the organisms by the neighbor-joining method using CVTree version 2 [13]. (**B**) Phylogenetic trees showing the relationship of *T. marneffei* with other molds and yeasts, inferred from the: (**i**) internal transcribed spacer (ITS) region; and (**ii**) RNA polymerase II second largest subunit gene (*rpb2*) sequence data by the maximum likelihood method with the substitution models: (**i**) Kimura 2-parameter (K2) with gamma-distributed rate variation (+G); and (**ii**) Tamura 3-parameter (T92) + G utilizing using MEGA 6.0.6 [14]. The scale bar indicates the estimated number of substitutions per base. *T. marneffei* is highlighted in bold and red color. All names and accession numbers are given as cited in the International Nucleotide Sequence Databases. For (**B**), numbers at nodes (expressed in percentage) indicate levels of bootstrap support calculated from 1000 replicates, and values lower than 60 are not shown.

Since *T. marneffei* exhibits a thermally dimorphic mode of growth, a first and foremost question that one will ask is whether it is phylogenetically more related to molds or yeasts. Based on phylogenetic analysis of all the coding sequences within the complete fungal mitogenomes, *T. marneffei* is found to be most closely related to other molds such as *Talaromyces*, *Penicillium*, and *Aspergillus* species (Figure 2A) [12]. When other DNA markers, including the ITS region and RNA polymerase II second largest subunit gene (*rpb2*), were used for analyzing the phylogeny of *T. marneffei*, results were found to be in line with those obtained using the mitochondrial genomes for analysis (Figure 2B). 

## 3. Sexual Stage

Traditionally, *T. marneffei* was believed to be an “asexual” fungus. In 2005, a report described *T. marneffei* as the potentially “most asexual fungus yet found” based on the fact that genetic diversity of *T. marneffei* was found to be generated principally due to mutation rather than recombination, leading to clonality with a highly significant linkage disequilibrium [15]. However, in our analysis of the *T. marneffei* PM1 genome, we found that all the genes involved in meiosis, except *HOP1*, as well as genes for pheromone receptors, enzymes which process pheromones, and proteins involved in pheromone response pathway in *Aspergillus nidulans* and *A. fumigatus* were present in the genome of *T. marneffei* PM1 [16]. Moreover, a putative *MAT-1* α box mating-type gene was also found and a putative *MAT-2* high-mobility group mating-type gene could be amplified from *T. marneffei* strains negative for *MAT-1* α box mating-type gene [16]. In addition, further experiments revealed that among 37 *T. marneffei* strains isolated from patients in Hong Kong, *MAT-1* α box and *MAT-2* high-mobility group mating-type genes could be respectively detected in 23 and 14 of the strains [16]. Based on these results, we concluded that *T. marneffei* is probably a heterothallic fungus that does not change mating type.

## 4. MP1 and Its Homologs

**Figure 3:**
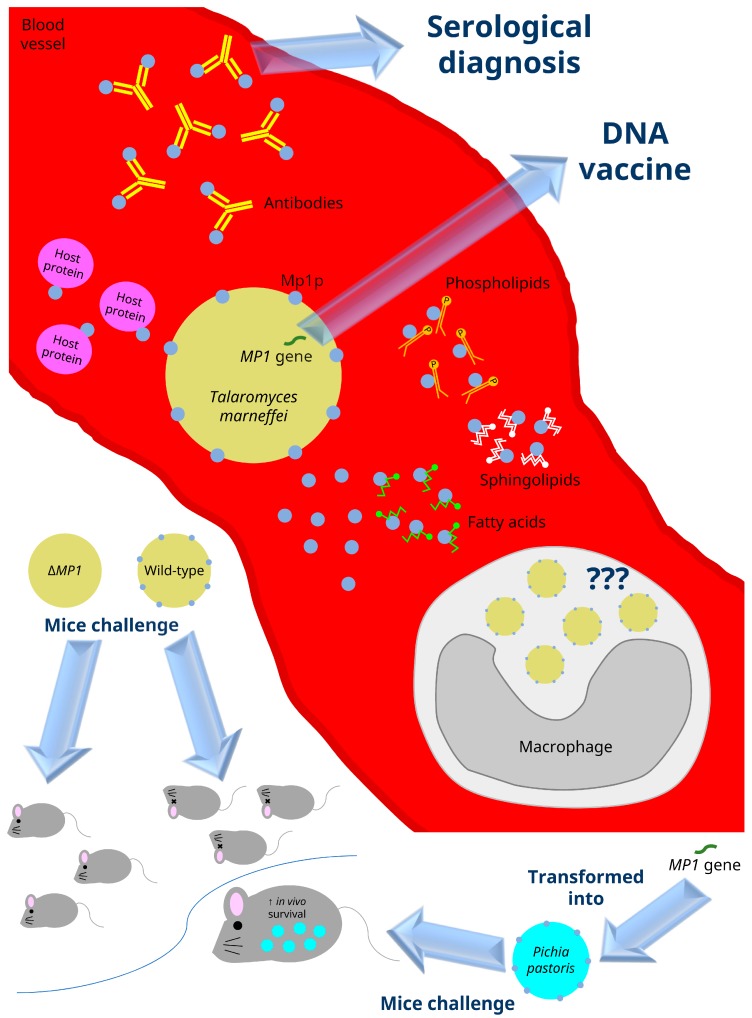
Virulence properties of the *Talaromyces marneffei* mannoprotein Mp1p. Mp1p is a cell wall protein and it could also be secreted out to the environment. It is highly antigenic and the detection of specific antibodies in patients’ sera against Mp1p constitutes one of the serological diagnostic methods for talaromycosis (penicilliosis). Mp1p also enhances the survival of the fungus inside macrophages, but how this protein mediates the evasion of host’s defense is not yet known. In addition, Mp1p is able to bind a variety of host factors, including proteins, fatty acids (such as arachidonic acid and palmitic acid), phospholipids, and sphingolipids. Wild-type *T. marneffei* is lethal to mice experimentally challenged with the fungus. However, when *MP1*-knock-out strain (Δ*MP1*) was used to infect experimental mice, none of the mice were killed. Moreover, when the *MP1* gene was transformed into an avirulent strain of *Pichia pastoris*, the expression of Mp1p in *P. pastoris* conferred the transformed fungus an improved survival rate inside experimental mice.

In 1998, the *MP1* gene was cloned and the Mp1p encoded by it was found to be a cell wall associated as well as secreted antigenic mannoprotein [17]. Subsequently, Mp1p as well as its homologs in *A. fumigatus* and *A. flavus* were found to be useful for serological diagnosis of *T. marneffei* (Figure 3) and the corresponding *Aspergillus* infections in patients [18,19,20,21]. In the *T. marneffei* PM1 genome, 13 additional Mp1p homologs were observed [17,22]. In contrast to Mp1p which possesses two lipid binding domains, these 13 homologs only have one lipid binding domain [22].

## 5. Virulence Properties

In our recent study, Mp1p was confirmed to be an important virulence factor of *T. marneffei* using a mouse model [22]. Specifically, all the balb/c mice died when they were injected with wild-type *T. marneffei* PM1, but none of the experimental mice died when challenged with *MP1* knock-out mutant (Figure 3). Notably, in contrast to Mp1p, all the other Mp1p homologs were not virulence factors as demonstrated by the same mouse model [22]. The virulence property of Mp1p is mediated through evading the host defense by enhancing its survival in host macrophages [22] (Figure 3). However, the exact molecular mechanism of Mp1p is still unknown. Although Mp1p was observed to be able to bind palmitic acid [23] and arachidonic acid [24] (Figure 3), its relationship to virulence was not shown in site-directed mutagenesis experiments. Moreover, our recent experiments revealed that in addition to these fatty acids, Mp1p is also able to bind a number of host proteins and phospholipids [25] (Figure 3), indicating that the molecular mechanism for its virulence property is probably more complicated than we thought.

## 6. Multilocus Sequence Typing

Since its first use in the late 1990s [26], multilocus sequence typing (MLST) is the state-of-the-art molecular typing method for bacteria. MLST involves PCR amplification and sequencing 5–7 housekeeping genes and the nucleotide sequences of the genes are used to determine the sequence type of the bacterial strain. For *T. marneffei*, when a set of housekeeping genes were sequenced for a number of strains, it was observed that all the sequences were identical in all the *T. marneffei* strains [27]. Therefore, the more lineage-specific *MP1* gene and its homologs were sequenced for a number of strains. Results showed that the sequences for five genes, including *MP1*, *MPLP4*, *MPLP7*, *MPLP10*, and *MPLP13*, particularly *MP1*, were variable; and therefore were used for constructing an MLST scheme for *T. marneffei* [27]. After sequencing these five loci in 44 *T. marneffei* strains recovered from patients in Hong Kong, this scheme was found to be exceedingly discriminatory, with a discriminatory power of 0.9884 [27]. However, the sequence types of the *T. marneffei* were found to be not related to the epidemiological parameters, such as age, sex, and HIV status of the patients [27]. 

## 7. Polyketide Synthases and Pigments

Polyketides are a diverse group of microorganism-produced secondary metabolites. Although non-essential, these metabolites include compounds (e.g., pigments, antibiotics, and mycotoxins) that provide survival advantages to the microbes. Polyketides are synthesized by polyketide synthases (PKS) which together constitute complex enzymatic systems. The *T. marneffei* PM1 genome contains 23 putative PKS genes and two putative PKS-non-ribosomal peptide synthase hybrid genes [11]. This diversity of intra-host PKS and PKS-related genes is much higher when compared with other pathogenic thermally dimorphic fungi, such as *Coccidioides immitis* and *Histoplasma capsulatum*, which only possess ten and one putative PKS genes, respectively [28]. Phylogenetic analysis revealed that these *T. marneffei* PKS genes were evenly distributed among the PKS genes of other fungi in the phylogenetic tree [29]. This suggested that such a huge PKS gene diversity in *T. marneffei* was not due to lineage-specific gene expansion through recent gene duplication [29]. 

Among these 23 PKS genes in the *T. marneffei* genome, the functions and their products of four were characterized. When the 23 PKS genes were knocked-down one by one, it was observed that the black pigment in the conidia was lost in one [30], the diffusible red pigment in the mycelial form was lost in another [31], and the yellow pigment in the mycelial form was lost in two other clones [28]. The black pigment is melanin and is a virulence factor mediated through resistance to hydrogen peroxide killing [30]. The yellow pigment consists of mitorubrinic acid and mitorubrinol [28]. Two PKS genes are sequentially used in the biosynthesis of these two compounds, and they are virulence factors of *T. marneffei* which have been shown to enhance the survival of the fungus in macrophages [28]. The diffusible red pigment is composed of a mixture of monascorubin- and rubropunctatin-amino acid conjugates [31]. The synthesis of monascorubin and rubropunctatin are controlled by the corresponding PKS genes and four additional genes downstream to it [31]. Recently, it was shown that the *sakA* gene of *T. marneffei*, a gene that is widely involved in asexual development, chitin deposition, oxidative and heat stresses tolerance, survival inside mouse and human macrophages, and yeast cell generation [32], also affects production of the red pigment [33]. The biology of these PKS genes and pigments in *T. marneffei*, including the biosynthetic pathway for red pigment production, has been recently reviewed by Tam et al. [29]. 

## 8. microRNA

MicroRNAs (miRNAs) are small non-coding endogenous RNAs that are important gene regulatory molecules. Mature miRNAs, which are about 22 nucleotides in length, down-regulate post-transcriptional gene expression of specific target genes by binding to complementary mRNAs at untranslated (UTR) regions or within the open-reading frames. miRNA-like small RNAs (milRNAs) have been detected in various fungi [34,35,36] including human pathogenic fungi such as *Cryptococcus neoformans* [37]. Recently, we have identified milRNAs in both growth phases of *T. marneffei*, which represents the first report of miRNAs in thermally dimorphic fungi [38]. Using high-throughput Illumina sequencing, 24 putative milRNA candidates, which were expressed more abundantly in hyphal cells than in yeast cells, were found. Three genes, *dcl-1*, *dcl-2*, and *qde-2*, encoding potential miRNA-processing proteins, namely Dicer-like proteins (dcl-1 and dcl-2) and Argonaute-like protein (qde-2), were also identified in the *T. marneffei* genome sequence [38]. 

Using *dcl-1*, *dcl-2*, and *qde-2* knock-out mutants, it has been shown that the expression of two milRNAs, PM-milR-M1 and PM-milR-M2, found only in hyphal cells, was dependent on *dcl-2* but not *dcl-1* or *qde-2*, supporting that *dcl-2* is involved in the biosynthesis of PM-milR-M1 and PM-milR-M2 milRNAs in *T. marneffei* [38]. Interestingly, *dcl-2* was also expressed more abundantly in hyphal cells than in yeast cells. Moreover, *dcl-2* of *T. marneffei* was phylogenetically more closely related to the homologs of the other pathogenic thermally dimorphic fungi, namely *Blastomyces dermatitidis*, *Coccidioides immitis*, *Histoplasma capsulatum*, and *Paracoccidioides brasiliensis*, than to those of other *Penicillium* and *Aspergillus* species, as shown by the closer clustering of *dcl-2* of *T. marneffei* to those of other thermally dimorphic fungi upon phylogenetic analysis. This is in contrast to phylogenetic analyses of the mitochondrial genome or house-keeping genes such as *rpb2* in which genes of *T. marneffei* are usually clustered with those of *Penicillium* and *Aspergillus* species (Figure 2). The findings suggest that *dcl-2* has co-evolved among the thermally dimorphic fungi instead of along species evolution [38]. Further studies may be conducted to explore for parallel pathways of milRNA processing and differential milRNAs expression in different growth phases in other thermally dimorphic fungi.

There is still a lot to be done to ascertain the function of miRNAs in *T. marneffei*. In our previous study, although potential gene targets were predicted for the different milRNA candidates [38], it remains to be determined if they are genuine targets of gene regulation for each miRNA. When a PM-milR-M1 knock-down strain was compared with the wild-type PM1 strain, the expression of three predicted gene targets, including the putative RanBP10 gene, the putative benzoate 4-monooxygenase cytochrome P450 gene, and a gene encoding a conserved hypothetical protein, were found to be upregulated by 1.7–3.8 folds [38]. However, the biological effects of such gene regulation remain unknown. Similar studies should also be performed to examine the role of other milRNAs in the regulation of their potential target genes in *T. marneffei*.

## 9. Transcriptome Profiling

Transcriptomics is the study of the full set of mRNAs expressed by a cell or a specific population of cells under a defined condition. Profiling of transcriptomes could usually be achieved by high-throughput DNA microarrays or by RNA sequencing employing next-generation sequencing technologies. Not only could transcriptome profiling inform the identity of each mRNA expressed by a specific cell or cell population, the quantity of each type of these mRNAs could also be revealed. Transcriptome profiling has been employed to give insights into the physiologies of different *T. marneffei* growth phases/forms. Using DNA microarrays, Lin et al. identified 1884 genes differentially expressed in yeast and hyphal cells of *T. marneffei* [39]. These genes could be grossly categorized into 18 different groups according to their biological functions. Moreover, 11 highly differential pathways were found. Notably, genes involved in the mitogen-activated protein kinase (MAPK) signaling and fatty acid metabolism pathways were expressed differentially between yeast and hyphal cells, suggesting that these two pathways may play important roles for *T. marneffei* to grow as yeast cells [39]. Subsequently in another microarray analysis, Pasricha et al. found that genes upregulated during early yeast phase development and throughout yeast growth included genes which are responsible for nutrient assimilation, in particular iron acquisition [40]. Improved iron uptake in yeast cells may help them survive the hostile environment inside host cells. In addition, genes involved in cell wall synthesis, such as the chitin synthase-encoding *chsE*, were also found upregulated in yeast cells. This implied that there is an increased production of chitin in yeast cells which may exert an important effect to host-pathogen interaction [40]. However, the limitation of these DNA microarray studies was that not all the transcripts were characterized. For example, in the first study, the design of the microarray panel only allowed identification of differential genes which were orthologous to genes of *Saccharomyces cerevisiae* with identified Gene Ontology (GO) annotations [39]; whereas in the second study the microarray panel was predicted to cover only 42% of the entire *T. marneffei* genome [40].

Different from the DNA microarray approach, we have previously attempted to characterize the transcriptome of *T. marneffei* using RNA sequencing [41]. It was revealed that there are a total of 11,042 predicted protein-coding genes in *T. marneffei*, which could be alternatively spliced to give 15,567 unique transcripts [41]. Of these, 1447 genes were over-expressed in yeast cells while another 1414 were under-expressed. Unexpectedly, heat-responsive genes were found not to be overexpressed in yeast cells, suggesting that *T. marneffei* may employ an alternative, unknown genetic regulatory strategy in response to temperature elevation which remains to be characterized. Moreover, it was also found that genes containing tandem repeat sequences (TRSs) were overexpressed in yeast cells. The expression products of these TRS-containing genes usually contain repeated amino acid residues, leading to polymorphism of these proteins among a population of *T. marneffei* strains and resulting in a variation of phenotypes. The diversity of phenotypes, particularly those related to adhesion, flocculation, and biofilm formation, may help *T. marneffei* yeast cells to disguise themselves and therefore better evade the immune response of the host [41]. On the other hand, another recent study by our group has identified *madsA* as a differential gene overexpressed during the transition from yeast cells to hyphal cells. *madsA* encodes a MADS-box transcription factor [42]. Overexpression of *madsA* in *T. marneffei* at 37 °C resulted in mycelium development, suggesting that *madsA* is responsible for the control of dimorphic transition from yeast to mold [42]. 

## 10. Proteome Profiling

Proteomics is the characterization of the whole set of proteins synthesized by an organism or a system, most commonly using two-dimensional polyacrylamide gel electrophoresis coupled with mass spectrometry (MS) for protein resolution and identification. The technique has been used for several purposes in studying *T. marneffei*, most often in an attempt to elucidate the mechanisms of dimorphic switching. Xi et al. have identified 26 proteins with differential expression in the yeast cells and hyphal cells [43]. Catalase-peroxidase, cytochrome P-450, Hsp90 and binding protein, as well as isocitrate lyase were expressed more abundantly in yeast cells, while poly(A) polymerase and SNF22 were expressed more abundantly in the hyphal cells. In another study, Chandler et al. identified different sets of proteins common or specific to the early development stages of the two growth phases [44]. Proteins with increased expression in the yeast development phase were found to be involved in cell wall biosynthesis, general metabolism, and heat-shock responses. In particular, the RanA protein, which is responsible for the regulation of mitosis and nuclear membrane transport, was hypothesized to be involved in the signaling mechanisms of dimorphism.

Using proteomic profiling, we have also previously identified glyceraldehyde-3-phosphate dehydrogenase (GAPDH) as an adhesion factor for *T. marneffei* conidial attachment [45]. Among the 12 identified proteins with differential expression in the culture supernatant from hyphal and yeast cells, GAPDH, which was up-regulated in hyphal cells, was subject to further adhesion assays. Adhesion of *T. marneffei* conidia to fibronectin and laminin as well as A549 pneumocytes was inhibited by recombinant GAPDH (rGAPDH) or anti-rGAPDH antibody in a dose-dependent manner, supporting the role of GAPDH in mediating conidial binding to human extracellular matrix proteins and pneumocytes, which may be important for the early establishment phase of infection in the lungs of patients with talaromycosis (penicilliosis) [45]. The application of proteomics in future studies may further shed lights in the pathogenesis and morphogenesis of this special fungus.

## 11. Concluding Remarks

As the life’s recipe cookbook of every single microbe in the world, the genome provides all the instructions for the creation of various phenotypes. In the current genomic era, genome sequencing can be achieved at a low cost using second-generation (e.g., 454 pyrosequencing and Solexa/Illumina technology) and recently third-generation (e.g., Pacific Biosciences Single Molecule, Real-Time (SMRT) sequencing platform, Ion Torrent semiconductor-based system, and Oxford Nanopore MinION/PromethION panels) sequencing technologies coupled with downstream sequence annotation as well as functional and comparative genomics work which could be efficaciously attained by complicated bioinformatics utilities along with transcriptomics and proteomics tools. In addition, facilitation of microbial metabolomics study by contemporary advanced techniques, such as ultra high performance liquid chromatography (UHPLC)—photodiode array detector (PDA)/electrospray ionization (ESI)—quadruple (Q)—time of flight (ToF) MS and nuclear magnetic resonance (NMR), has greatly improved the characterization of biochemical and metabolic fingerprints of microorganisms. In the last decade or so, we have used all these state-of-the-art genomic, transcriptomic, proteomic, and metabolomic lenses to examine how *T. marneffei* adapts to its environments, evade our immune system, and cause disease in humans at different angles. These studies have revealed potential novel targets for preventive and therapeutic interventions. Based on these various omics data, future work could be performed to induce the development of the sexual stage of the fungus, or to elucidate the functions and uses of other PKS genes, etc. to advance our understanding on this fungal pathogen. The ultimate goal should aim at utilizing these data for the design of novel antifungal agents and vaccines for the control of *T. marneffei* infection.

## References

[1] Capponi M., Sureau P., Segretain G. (1956). Pénicilliose de *Rhizomys sinensis*. Bull. Soc. Pathol. Exot..

[2] Deng Z., Yun M., Ajello L. (1986). Human penicilliosis marneffei and its relation to the bamboo rat (*Rhizomys pruinosus*). J. Med. Vet. Mycol..

[3] Ajello L., Padhye A.A., Sukroongreung S., Nilakul C.H., Tantimavanic S. (1995). Occurrence of *Penicillium marneffei* infections among wild bamboo rats in Thailand. Mycopathologia.

[4] Chariyalertsak S., Vanittanakom P., Nelson K.E., Sirisanthana T., Vanittanakom N. (1996). *Rhizomys sumatrensis* and *Cannomys badius*, new natural animal hosts of *Penicillium marneffei*. J. Med. Vet. Mycol..

[5] Samson R.A., Yilmaz N., Houbraken J., Spierenburg H., Seifert K.A., Peterson S.W., Varga J., Frisvad J.C. (2011). Phylogeny and nomenclature of the genus *Talaromyces* and taxa accommodated in *Penicillium* subgenus *Biverticillium*. Stud. Mycol..

[6] Vanittanakom N., Cooper C.R., Fisher M.C., Sirisanthana T. (2006). *Penicillium marneffei* infection and recent advances in the epidemiology and molecular biology aspects. Clin. Microbiol. Rev..

[7] Nittayananta W. (1999). Penicilliosis marneffei: Another AIDS defining illness in Southeast Asia. Oral Dis..

[8] Deng Z., Ribas J.L., Dean W.G., Connor D.H. (1988). Infections caused by *Penicillium marneffei* in China and Southeast Asia: Review of eighteen published cases and report of four more chinese cases. Rev. Infect. Dis..

[9] Chan J.F.W., Chan T.S.Y., Gill H., Lam F.Y.F., Trendell-Smith N.J., Sridhar S., Tse H., Lau S.K.P., Hung I.F.N., Yuen K.-Y. (2015). Disseminated infections with *Talaromyces marneffei* in non-AIDS patients given monoclonal antibodies against CD20 and kinase inhibitors. Emerg. Infect. Dis..

[10] Chan J.F.W., Lau S.K.P., Yuen K.-Y., Woo P.C.Y. (2016). *Talaromyces* (*Penicillium*) *marneffei* infection in non-HIV-infected patients. Emerg. Microbes Infect..

[11] Woo P.C.Y., Lau S.K.P., Liu B., Cai J.J., Chong K.T.K., Tse H., Kao R.Y.T., Chan C.-M., Chow W.-N., Yuen K.-Y. (2011). Draft genome sequence of *Penicillium marneffei* strain PM1. Eukaryot. Cell.

[12] Tam E.W.T., Tsang C.-C., Lau S.K.P., Woo P.C.Y. (2016). Comparative mitogenomic and phylogenetic characterization on the complete mitogenomes of *Talaromyces* (*Penicillium*) *marneffei*. Mitochondrial DNA B Resour..

[13] Xu Z., Hao B. (2009). CVTree update: A newly designed phylogenetic study platform using composition vectors and whole genomes. Nucleic Acids Res..

[14] Tamura K., Stecher G., Peterson D., Filipski A., Kumar S. (2013). MEGA6: Molecular Evolutionary Genetics Analysis Version 6.0. Mol. Biol. Evol..

[15] Fisher M.C., Hanage W.P., de Hoog S., Johnson E., Smith M.D., White N.J., Vanittanakom N. (2005). Low effective dispersal of asexual genotypes in heterogeneous landscapes by the endemic pathogen *Penicillium marneffei*. PLoS Pathog..

[16] Woo P.C.Y., Chong K.T.K., Tse H., Cai J.J., Lau C.C.Y., Zhou A.C., Lau S.K.P., Yuen K.-Y. (2006). Genomic and experimental evidence for a potential sexual cycle in the pathogenic thermal dimorphic fungus *Penicillium marneffei*. FEBS Lett..

[17] Cao L., Chan C.-M., Lee C., Wong S.S.Y., Yuen K.-Y. (1998). MP1 encodes an abundant and highly antigenic cell wall mannoprotein in the pathogenic fungus *Penicillium marneffei*. Infect. Immun..

[18] Cao L., Chan K.-M., Chen D., Vanittanakom N., Lee C., Chan C.-M., Sirisanthana T., Tsang D.N.C., Yuen K.-Y. (1999). Detection of cell wall mannoprotein Mp1p in culture supernatants of *Penicillium marneffei* and in sera of penicilliosis patients. J. Clin. Microbiol..

[19] Yuen K.-Y., Chan C.-M., Chan K.-M., Woo P.C.Y., Che X.-Y., Leung A.S.P., Cao L. (2001). Characterization of AFMP1: A novel target for serodiagnosis of *Aspergillosis*. J. Clin. Microbiol..

[20] Woo P.C.Y., Chan C.-M., Leung A.S.P., Lau S.K.P., Che X.-Y., Wong S.S.Y., Cao L., Yuen K.-Y. (2002). Detection of cell wall galactomannoprotein AFMP1P in culture supernatants of *Aspergillus fumigatus* and in sera of aspergillosis patients. J. Clin. Microbiol..

[21] Woo P.C.Y., Chong K.T.K., Leung A.S.P., Wong S.S.Y., Lau S.K.P., Yuen K.-Y. (2003). AFLMP1 encodes an antigenic cell wall protein in *Aspergillus flavus*. J. Clin. Microbiol..

[22] Woo P.C.Y., Lau S.K.P., Lau C.C.Y., Tung E.T.K., Chong K.T.K., Yang F., Zhang H., Lo R.K.C., Cai J.-P., Au-Yeung R.K.H. (2016). Mp1p is a virulence factor in *Talaromyces* (*Penicillium*) *marneffei*. PLoS Negl. Trop. Dis..

[23] Liao S., Tung E.T.K., Zheng W., Chong K., Xu Y., Dai P., Guo Y., Bartlam M., Yuen K.-Y., Rao Z. (2010). Crystal structure of the Mp1p ligand binding domain 2 reveals its function as a fatty acid-binding protein. J. Biol. Chem..

[24] Sze K.-H., Lam W.-H., Zhang H., Ke Y.-H., Tse M.-K., Woo P.C.Y., Lau S.K.P., Lau C.C.Y., Cai J.-P., Tung E.T.K. (2017). *Talaromyces marneffei* Mp1p Is a virulence factor that binds and sequesters a key proinflammatory lipid to dampen host innate immune response. Cell Chem. Biol..

[25] Woo P.C.Y., Tsang C.-C., Xue S., Yang F., Tan Y.-P., Cai J.-P., Kok K.-H., Yuen K.-Y., Lau S.K.P. (2017). *Talaromyces marneffei* Mp1p binds a variety of proteins, sphingolipids and phospholipids: Implication on virulence mechanism.

[26] Maiden M.C.J., Bygraves J.A., Feil E., Morelli G., Russell J.E., Urwin R., Zhang Q., Zhou J., Zurth K., Caugant D.A. (1998). Multilocus sequence typing: A portable approach to the identification of clones within populations of pathogenic microorganisms. Proc. Natl. Acad. Sci. USA.

[27] Woo P.C.Y., Lau C.C.Y., Chong K.T.K., Tse H., Tsang D.N.C., Lee R.A., Tse C.W.S., Que T.-L., Chung L.M.W., Ngan A.H.Y. (2007). MP1 homologue-based multilocus sequence system for typing the pathogenic fungus *Penicillium marneffei*: A novel approach using lineage-specific genes. J. Clin. Microbiol..

[28] Woo P.C.Y., Lam C.-W., Tam E.W.T., Leung C.K.F., Wong S.S.Y., Lau S.K.P., Yuen K.-Y. (2012). First discovery of two polyketide synthase genes for mitorubrinic acid and mitorubrinol yellow pigment biosynthesis and implications in virulence of *Penicillium marneffei*. PLoS Negl. Trop. Dis..

[29] Tam E.W.T., Tsang C.-C., Lau S.K.P., Woo P.C.Y. (2015). Polyketides, toxins and pigments in *Penicillium marneffei*. Toxins.

[30] Woo P.C.Y., Tam E.W.T., Chong K.T.K., Cai J.J., Tung E.T.K., Ngan A.H.Y., Lau S.K.P., Yuen K.-Y. (2010). High diversity of polyketide synthase genes and the melanin biosynthesis gene cluster in *Penicillium marneffei*. FEBS J..

[31] Woo P.C.Y., Lam C.-W., Tam E.W.T., Lee K.-C., Yung K.K.Y., Leung C.K.F., Sze K.-H., Lau S.K.P., Yuen K.-Y. (2014). The biosynthetic pathway for a thousand-year-old natural food colorant and citrinin in *Penicillium marneffei*. Sci. Rep..

[32] Nimmanee P., Woo P.C.Y., Kummasook A., Vanittanakom N. (2015). Characterization of *sakA* gene from pathogenic dimorphic fungus *Penicillium marneffei*. Int. J. Med. Microbiol..

[33] Nimmanee P., Tam E.W.T., Woo P.C.Y., Vanittanakom P., Vanittanakom N. (2017). Role of the *Talaromyces marneffei* (*Penicillium marneffei*) *sakA* gene in nitrosative stress response, conidiation and red pigment production. FEMS Microbiol. Lett..

[34] Lee H.-C., Li L., Gu W., Xue Z., Crosthwaite S.K., Pertsemlidis A., Lewis Z.A., Freitag M., Selker E.U., Mello C.C. (2010). Diverse pathways generate microRNA-like RNAs and Dicer-independent small interfering RNAs in fungi. Mol. Cell.

[35] Zhou J., Fu Y., Xie J., Li B., Jiang D., Li G., Cheng J. (2012). Identification of microRNA-like RNAs in a plant pathogenic fungus *Sclerotinia sclerotiorum* by high-throughput sequencing. Mol. Genet. Genom..

[36] Zhou Q., Wang Z., Zhang J., Meng H., Huang B. (2012). Genome-wide identification and profiling of microRNA-like RNAs from *Metarhizium anisopliae* during development. Fungal Biol..

[37] Jiang N., Yang Y., Janbon G., Pan J., Zhu X. (2012). Identification and functional demonstration of miRNAs in the fungus *Cryptococcus neoformans*. PLoS ONE.

[38] Lau S.K.P., Chow W.-N., Wong A.Y.P., Yeung J.M.Y., Bao J., Zhang N., Lok S., Woo P.C.Y., Yuen K.-Y. (2013). Identification of microRNA-like RNAs in mycelial and yeast phases of the thermal dimorphic fungus *Penicillium marneffei*. PLoS Negl. Trop. Dis..

[39] Lin X., Ran Y., Gou L., He F., Zhang R., Wang P., Dai Y. (2012). Comprehensive transcription analysis of human pathogenic fungus *Penicillium marneffei* in mycelial and yeast cells. Med. Mycol..

[40] Pasricha S., Payne M., Canovas D., Pase L., Ngaosuwankul N., Beard S., Oshlack A., Smyth G.K., Chaiyaroj S.C., Boyce K.J. (2013). Cell-type–specific transcriptional profiles of the dimorphic pathogen *Penicillium marneffei* reflect distinct reproductive, morphological, and environmental demands. G3 (Bethesda).

[41] Yang E., Wang G., Woo P.C.Y., Lau S.K.P., Chow W.-N., Chong K.T.K., Tse H., Kao R.Y.T., Chan C.-M., Che X. (2013). Unraveling the molecular basis of temperature-dependent genetic regulation in *Penicillium marneffei*. Eukaryot. Cell.

[42] Yang E., Chow W.-N., Wang G., Woo P.C.Y., Lau S.K.P., Yuen K.-Y., Lin X., Cai J.J. (2014). Signature gene expression reveals novel clues to the molecular mechanisms of dimorphic transition in *Penicillium marneffei*. PLoS Genet..

[43] Xi L., Xu X., Liu W., Li X., Liu Y., Li M., Zhang J., Li M. (2007). Differentially expressed proteins of pathogenic *Penicillium marneffei* in yeast and mycelial phases. J. Med. Microbiol..

[44] Chandler J.M., Treece E.R., Trenary H.R., Brenneman J.L., Flickner T.J., Frommelt J.L., Oo Z.M., Patterson M.M., Rundle W.T., Valle O.V. (2008). Protein profiling of the dimorphic, pathogenic fungus, *Penicillium marneffei*. Proteome Sci..

[45] Lau S.K.P., Tse H., Chan J.S.Y., Zhou A.C., Curreem S.O.T., Lau C.C.Y., Yuen K.-Y., Woo P.C.Y. (2013). Proteome profiling of the dimorphic fungus *Penicillium marneffei* extracellular proteins and identification of glyceraldehyde-3-phosphate dehydrogenase as an important adhesion factor for conidial attachment. FEBS J..

